# *Besnoitia besnoiti*-induced neutrophil clustering and neutrophil extracellular trap formation depend on P2X1 purinergic receptor signaling

**DOI:** 10.3389/fimmu.2023.1244068

**Published:** 2023-10-03

**Authors:** Gabriel Espinosa, Iván Conejeros, Lisbeth Rojas-Barón, Carlos Rodrigo Hermosilla, Anja Taubert

**Affiliations:** Institute of Parasitology, Justus Liebig University Giessen, Giessen, Germany

**Keywords:** PMN, *Besnoitia besnoiti*, NET formation, ATP, purinergic receptors, immunometabolism

## Abstract

Bovine besnoitiosis is a re-emerging cattle disease caused by the cyst-forming apicomplexan parasite *Besnoitia besnoiti*. Neutrophil extracellular trap (NET) formation represents an efficient innate immune mechanism of polymorphonuclear neutrophils (PMN) against apicomplexan parasites, including *B. besnoiti*. PMN purinergic signaling was proposed as a critical factor for NET formation. One important purinergic ligand is ATP, which is recognized as a danger signal and released into the extracellular space acting as an autocrine/paracrine signaling molecule. ATP-driven effects on PMN via the nucleotide P2 receptor family include chemotaxis, reactive oxygen species (ROS) production, and NET formation. So far, data on both PMN ATP concentrations and the role of ATP as a key modulator of purinergic signaling in *B. besnoiti* tachyzoite-triggered bovine NETosis is scarce. Current data showed that *B. besnoiti* tachyzoite exposure to bovine PMN neither changed total PMN ATP nor extracellular ATP quantities even though it significantly triggered NET formation. Moreover, *B. besnoiti* tachyzoite-exposed PMN revealed enhanced oxygen consumption rates (OCR) as quantified by the Seahorse metabolic analyzer. Exogenous supplementation of ATP or non-hydrolizable ATP (ATPγS) led to increased extracellular acidification rates (ECAR) but failed to alter tachyzoite-induced oxidative responses (OCR) in exposed PMN. In addition, exogenous supplementation of ATPγS, but not of ATP, boosted *B. besnoiti* tachyzoite-induced anchored NET formation. Referring to purinergic signaling, *B. besnoiti* tachyzoite-triggered anchored NET formation revealed P2X1 purinergic as receptor-dependent since it was blocked by the P2X1 inhibitor NF449 at an IC_50_ of 1.27 µM. In contrast, antagonists of P2Y2, P2Y6, P2X4, and P2X7 purinergic receptors all failed to affect parasite-driven NETosis. As an interesting finding, we additionally observed that *B. besnoiti* tachyzoite exposure induced PMN clustering in a P2X1-dependent manner. Thus, we identified P2X1 purinergic receptor as a pivotal molecule for both *B. besnoiti* tachyzoite*-*induced PMN clustering and anchored NET formation.

## 1 Introduction

*Besnoitia besnoiti* is a cyst-forming apicomplexan parasite closely related to *Toxoplasma gondii* and *Neospora caninum*. It is the causal agent of bovine besnoitiosis, a severe but mainly non-fatal disease with significant economic impact in many countries in Africa, Asia, and Europe (1, 2). In 2010, the disease was classified as an emerging disease by the European Food Safety Authority (EFSA). Typical clinical signs of acute besnoitiosis are pyrexia, photophobia, lacrimation, tachypnoe, acute orchitis, and subcutaneous edema. In chronically infected cattle, parasitic cysts develop in various organs, including skin, vascular walls, sclera, and other non-intestinal mucous membranes. Typically, sklerodermia with skin thickening, abrasion, and alopecia occurs. Moreover, skin alterations of the udder impair proper milking. Chronic *B. besnoiti* infections of bulls may cause orchitis leading to transient or permanent infertility (3). *B. besnoiti* can be mechanically transmitted by hematophagous tabanids (*Tabanus* spp.) and biting muscids like *Stomoxys calcitrans*; the role of transmission via mating remains unclear so far. To date, the definitive host of *B. besnoiti* is unknown (3, 4).

Referring to immunological responses in *B. besnoiti* tachyzoite*-*infected cattle, (immune) histochemical studies show leukocyte infiltration in the dermis of affected animals (5), indicating that early host defense responses are important for disease progression. Polymorphonuclear neutrophils (PMN) represent the most abundant leukocyte population in the blood and are amongst the first innate immune cell types responding against infection. PMN also participate in the resolution of inflammation and wound healing (6). In general, PMN react against a variety of invasive pathogens, such as bacteria, viruses, fungi, protozoan, and metazoan parasites. Different PMN-derived effector mechanisms have been demonstrated, including the release of immunomodulatory molecules [e.g., cytokines and chemokines], phagocytosis, production of reactive oxygen species (ROS), and release of neutrophil extracellular traps (NETs) (6–9). Referring to the latter mechanism, NETs are typically composed of DNA, histones, and microbicidal peptides and bear the capacity to kill certain pathogens like bacteria (10) or to limit pathogen spread in infected organisms by local trapping activities (11). NETs are released via a cell death process known as NETosis (12). NETosis is either linked to a nicotinamide adenine dinucleotide phosphate (NADPH) oxidase (NOX)-dependent or NOX-independent cellular mechanism, which triggers nuclear chromatin decondensation facilitated by protein arginine deiminase 4 (PAD4)-mediated citrullination of histones, fast actin cytoskeleton disassembly, microtubules remodeling, and shedding of plasma membrane microvesicles containing granules and cytosolic components. Following this, enzymes like neutrophil elastase (NE) and myeloperoxidase (MPO) translocate to the nucleus and fuse with chromatin. Finally, PMN membrane disintegration is either mediated by enhanced ROS production or by lytic proteins like gasdermin D mediating membrane pore formation and subsequent NET extrusion into the extracellular matrix (11–17).

The protozoan parasite-driven NET release is reported for *Eimeria bovis* (18, 19)*, Eimeria arloingi* (20)*, Toxoplasma gondii* (21–23)*, Cryptosporidium parvum* (24)*, Neospora caninum* (25–27)*, Trypanosoma brucei brucei* (28), and *B. besnoiti* (29–32). Notably, *B. besnoiti* tachyzoite*-*driven NETosis seemed stage-independent since bradyzoites were also proven to trigger NETosis in bovine PMN (33). Referring to signaling cascades upstream of *B. besnoiti* tachyzoite*-*driven NET formation, tachyzoite exposure to PMN induced the phosphorylation of AMP-activated kinase α (AMPKα), and a positive correlation between LC3B-positive PMN and NET formation is described (31). More recently, metabolic responses of *B. besnoiti* tachyzoite-exposed bovine PMN revealed enhanced catabolism of glucose and serine, accompanied by increased glutamate production (32). Secondary metabolites in *B. besnoiti* tachyzoite*-*exposed PMN appeared pivotal for NET formation since chemical inhibition of lactate release, pyruvate dehydrogenase, α-ketoglutarate dehydrogenase, and transketolase significantly affected cell-free NET formation, thereby indicating a key role of pyruvate- and lactate-mediated metabolic pathways. Interestingly, tachyzoite-induced NET formation was also significantly blocked by treatments with oligomycin A (inhibitor of ATP synthase) and NF449 (purinergic receptor P2X1 antagonist), suggesting a key role of ATP-related responses in tachyzoite-driven NETosis (32).

Intracellular ATP represents the main source of energy driving almost all cell functions. However, ATP also plays a key role in purinergic signaling. Hence, stressed cells release ATP as a danger and ˝find me˝ signal, thereby guiding the migration of purinergic receptor-expressing leukocytes (e.g., PMN) which then respond to extracellular ATP and related nucleotides. In general, three major families of purinergic receptors are described based on their pharmacological and structural properties. P2X receptors function as ATP-gated ion channels that facilitate the influx of extracellular cations, including calcium. In contrast, P2Y receptors represent G protein-coupled receptors (GPCRs), which recognize ATP and several other nucleotides including ADP, UTP, UDP, and UDP-glucose. Moreover, P1 receptors are also GPCRs that recognize adenosine (34–37). Purinergic signaling in PMN regulates critical PMN functions via paracrine and autocrine (feedback) mechanisms. For example, the release of ATP via pannexin-1 (PANX1) channels to the extracellular environment regulates PMN chemotaxis, rolling/adhesion/transmigration, ROS generation, NETosis, and apoptosis (36, 38–43).

The aim of the current work was to analyze the role of purinergic signaling and the metabolic state of PMN in *B. besnoiti* tachyzoite-induced NET formation. Early PMN activation was studied via oxygen consumption (OCR) and extracellular acidification rates (ECAR) using the Seahorse technology. Moreover, the role of selected molecules of the purinergic signaling cascade in relevant PMN functions like clustering and NET formation was studied.

## 2 Materials and methods

### 2.1 Ethics statement

This study was performed in accordance with the Justus Liebig University Giessen Animal Care Committee Guidelines. Protocols were approved by the Ethics Commission for Experimental Animal Studies of the Federal State of Hesse (Regierungspräsidium Giessen; GI 18/10 Nr. V 2/2022; JLU-No. 0002_V) and are in accordance with European Animal Welfare Legislation: ART13TFEU and currently applicable German Animal Protection Laws.

### 2.2 Host cell culture and *B. besnoiti* tachyzoite maintenance

All experiments of the current study were performed with tachyzoite stages of the apicomplexan parasite *B. besnoiti* (strain Bb-Evora04). Madin-Darby bovine kidney (MDBK) cells were used as host cells for *B. besnoiti* tachyzoite in *in vitro* production. MDBK cell layers were cultured in 75 cm^2^ plastic tissue culture flasks (Greiner) at 37°C/5% CO_2_ atmosphere using RPMI 1640 (Sigma-Aldrich) cell culture medium supplemented with 5% fetal bovine serum (FBS, 10270-106, Gibco) and 1% penicillin/streptomycin (both 500 mg/ml, P4333, Sigma-Aldrich). MDBK cell layers were infected at 80% confluency with 2.4×10^7^ *B. besnoiti* tachyzoites. Tachyzoites released from host cells were collected from the supernatants, filtered through a 5 μm syringe filter (Merck Millipore), washed, and pelleted (400×g, 12 min) prior to re-suspension in RPMI 1640 cell culture medium (supplemented with 5% FBS and 1% penicillin/streptomycin). Tachyzoite numbers were determined in a Neubauer chamber, and parasite stages were placed at 37°C/5% CO_2_ atmosphere for further experimental use.

### 2.3 Bovine PMN isolation

Healthy adult dairy cows served as blood donors. Animals were bled by puncturing the jugular vein, and peripheral blood was collected in heparinized sterile plastic tubes (Kabe Labortechnik). A measurement of 20 mL of heparinized blood was re-suspended in 20 mL sterile PBS with 0.02% EDTA (CarlRoth), carefully layered on top of 12 ml Histopaque-1077 separating solution (density = 1.077 g/L; 10771, Sigma-Aldrich) and centrifuged (800×g, 45 min) without brake. After removal of plasma and peripheral blood mononuclear cells, the cell pellet was suspended in 20 mL of lysis buffer (5.5 mM NaH_2_PO_4_, 10.8 mM KH_2_PO_4_, and pH 7.2) and gently mixed for 60 s to lyse erythrocytes. Osmolarity was rapidly restored by the addition of 10 mL hypertonic buffer (462 mM NaCl, 5.5 mM NaH_2_PO_4_, 10.8 mM KH_2_PO_4_, and pH 7.2) and 10 mL of Hank’s balanced salt solution (14065-049, Gibco). The lysis step was repeated twice until no erythrocytes were visible. PMN were then suspended in 5 mL of HBSS, counted in a Neubauer chamber, and allowed to rest on ice for 30 min prior to any experimental use.

### 2.4 Scanning electron microscopy

Bovine PMN were co-cultured at 37°C and 5% CO_2_ with *B. besnoiti* tachyzoites at a 1:4 ratio for 60 min on 10 mm coverslips (Thermo Fisher Scientific) pre-coated with 0.01% poly-L-lysine (Sigma-Aldrich). After incubation, cells were fixed in 2.5% glutaraldehyde (Merck), post-fixed in 1% osmium tetroxide (Merck), washed in distilled water, dehydrated, critical point dried by CO_2_ treatment, and sputtered with gold. Finally, all samples were analyzed via a Philips XL30 scanning electron microscope at the Institute of Anatomy and Cell Biology, Justus Liebig University Giessen, Germany.

### 2.5 Immunofluorescence microscopy

Bovine PMN were co-cultured with *B. besnoiti* tachyzoites at a 1:6 ratio for 4 h (37°C, 5% CO_2_ atmosphere) on fibronectin- (2.5 ug/mL) pretreated coverslips (15 mm diameter, Thermo Fisher Scientific), fixed in 4% paraformaldehyde (Merck), and stored at 4°C until further use. For NET visualization, DAPI (Fluoromount G, ThermoFisher, 495952) was used to stain DNA. Anti-histone (clone TNT-1, 1:1000; Merck Millipore #MAB3864, Darmstadt, Germany) and anti-NE (AB68672, 1:1000, Abcam, Cambridge, UK) antibodies were used to detect respective proteins on NET structures. In addition, an in-house hyperimmune serum against *B. besnoiti* was used (1:100) to stain tachyzoite stages. For antibody-related reactions, fixed samples were washed three times with PBS, blocked with 1% bovine serum albumin (BSA, Sigma-Aldrich, Steinheim, Germany, 30 min, RT), and incubated in corresponding primary antibody solutions (1 h, RT). After three washings in PBS, samples were incubated in secondary antibody solutions (Alexa Fluor 488 goat anti-mouse IgG or Alexa Fluor 405 goat anti-rabbit IgG, both Life Technologies, Eugene, USA, 60 min, 1:1000, RT). Finally, samples were washed three times in PBS and mounted in an anti-fading buffer (Fluoromount G, ThermoFisher, 495952). Image acquisition was performed in a Ti2-A inverted microscope (Nikon) equipped with a white LED epifluorescence lamp using the NIS-Elements v 5.11 software (Nikon) and applying identical brightness and contrast conditions within the datasets of each biological experiment.

### 2.6 Quantification of “cell-free” and “anchored” NETs

Bovine PMN suspended in RPMI 1640 medium were confronted with *B. besnoiti* tachyzoites (4 h, 37°C, 5% CO_2_) at a PMN/tachyzoite ratio of 1:6 (2×10^5^ PMN:1.2×10^6^ tachyzoites, 96-well format) in the presence of non-modified ATP (P1132, Promega, USA), non-hydrolyzable ATP (ATPγS; 0.05-50 µM; 4080, Tocris, UK), or purinergic receptor antagonists (see **Table 1**) at a concentration range of 0.1-100 µM. After incubation, sample supernatants were analyzed for “cell-free” NETs. The remaining cells at the well bottoms were estimated for “anchored” NETs according to (44). Therefore, picogreen (Invitrogen, Eugene, USA, 1:200 dilution in 10 mM Tris base buffered with 1 mM EDTA, 50 μl/well) was added to each supernatant or pellet sample. Extracellular DNA was quantified by picogreen-derived fluorescence intensities using an automated microplate reader (Varioskan, Thermo Scientific) at 484 nm excitation/520 nm emission as described elsewhere (26, 30).

**Table 1:** Purinergic receptor antagonists.

Receptor	Antagonist	Cat. number (Company)
P2Y1	MRS 2179	0900 (Tocris)
P2Y6	MRS 2578	2146 (Tocris)
P2X1	NF449	1391 (Tocris)
P2X4	5-BDBD	3579 (Tocris)
P2X7	AZ10606120	3323 (Tocris)

### 2.7 Measurement of total and extracellular ATP concentrations

Bovine PMN of 1×10^6^ from three blood donors were confronted with *B. besnoiti* tachyzoites (1:6 ratio) in HBSS 1× (14065-049, Gibco) and incubated at 37°C/5% CO_2_ for both 15 s and 15 min. For positive controls, cells were stimulated with PMA/ionomycin (100 nM/5 µM) and HBSS supplemented with 1 M NaCl. After incubation on ice for 5 min, cells were pelleted (600 × g, 5 min) and supernatants were recovered for extracellular ATP quantification using an ATP Determination kit (A22066; Invitrogen) according to the manufacturer’s instructions. For the estimation of PMN total ATP concentration, whole cell pellets were analyzed by the CellTiter-Glo luminescent Cell viability assay (G7571, Promega) following the manufacturer’s instructions. All samples were analyzed by luminometry using an automated reader (Luminoskan Flash).

### 2.8 Quantification of PMN oxygen consumption rates and extracellular acidification rates

Activation of bovine PMN was monitored using the Seahorse XF analyzer (Agilent). Briefly, 1×10^6^ PMN from three blood donors were pelleted [500 × g, 10 min, room temperature (RT)]. The cell pellets were re-suspended in 0.25 ml of XF assay medium (Agilent) supplemented with 2 mM of _L_-glutamine, 1 mM pyruvate, and 10 mM glucose. A total of 2×10^5^ cells were gently placed in each well of an eight-well XF analyzer plate (Agilent) pre-coated for 30 min with 0.001% poly-_L_-lysine (Sigma-Aldrich). Then, the XF assay medium (Agilent) was adjusted to 180 μl total volume per well and cells were incubated at 37°C without CO_2_ supplementation for 45 min before Seahorse measurements. ATP, ATPγS (0.05-50 μM), and *B. besnoiti* (1.2×10^6^ tachyzoites) were suspended in an XF assay medium and supplemented to the cells via instrument-own injection ports after baseline measurements. The total assay duration was 240 min. Background subtraction and determination of OCR/ECAR registries were performed by using the Seahorse Agilent analytics platform (https://seahorseanalytics.agilent.com).

### 2.9 Assessment of NF449-mediated effects on PMN viability by flow cytometry

In order to determine potential NF449-driven effects on PMN apoptosis and necrosis rates, PMN suspensions (2×10^5^/200 µl HBSS) were placed into 5 mL polystyrene round-bottom tubes (352052, Falcon, Corning USA) and treated with NF449 (10-100 µM) for 4 h at 37°C/5%CO_2_. PMN were then pelleted (600 x g, 8 min) and stained using the Annexin V-FITC/PI double staining apoptosis detection kit (ab14085, Abcam) following the manufacturer´s instructions and analyzed with a BD Accuri C6 Plus Flow Cytometer (BD, USA). FlowJo v10.8.1 software was used to determine the apoptosis/necrosis rate.

### 2.10 Statistical analysis

All experiments were repeated at least three times. Statistical significance was defined by a *p*-value < 0.05. The *p*-values were determined by applying the following analyses: unpaired two-way t-tests were used for Seahorse-based metabolic measurements. Unpaired two-way t-tests and one-way ANOVA followed by Tukey´s multiple comparisons test were applied to ATP quantification experiments. One-way ANOVA followed by a Dunnett´s multiple comparison test was used to analyze extracellular DNA quantification experiments. Kruskal Wallis´s followed by Dunn´s multiple comparisons test was applied to extracellular DNA quantification assays using purinergic receptors inhibitors. One-way ANOVA followed by Tukey´s multiple comparisons test was used to analyze clustering experiments. Bar graphs represent the mean ± SD, and statistical analysis was generated using Graph Pad software (v. 7.03).

## 3 Results

### 3.1 *B. besnoiti* tachyzoite-exposed bovine PMN release NETs entrapping tachyzoites

**Figure 1:**
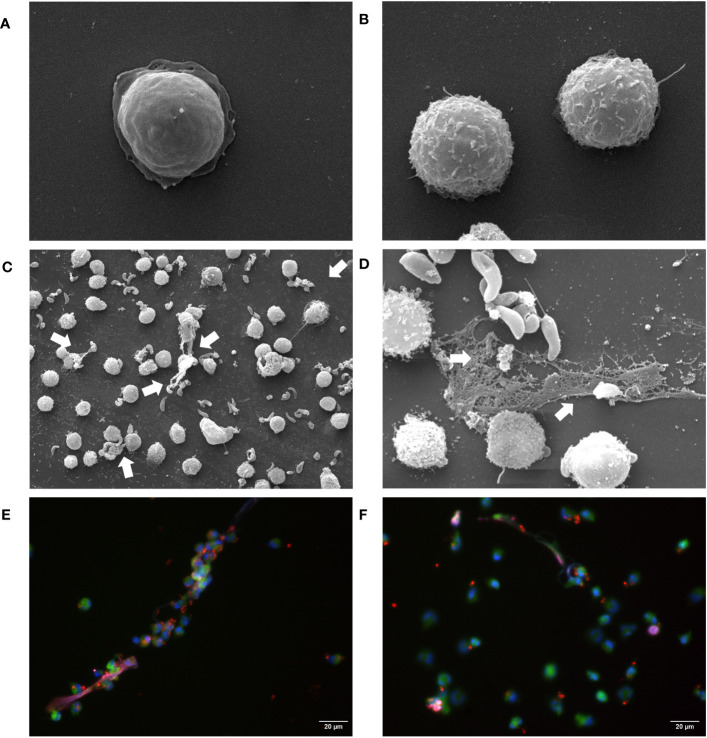
*B. besnoiti* tachyzoites are trapped in released NETs. **(A, B)** Unstimulated PMN. NETs defined as extracellular chromatin filaments containing proteins like neutrophil elastase and histone H1 formed a network in contact with *B. besnoiti* tachyzoites, as visualized via SEM **(C, D)** and immunostaining **(E, F)**. White arrows indicate entrapped *B. besnoiti* tachyzoites. Co-localization of DNA (DAPI, blue); *B. besnoiti* tachyzoites (red); Histone H1 clone TNT-1 staining (pink) and neutrophil elastase (NE, green).

To confirm the functional capacity of *B. besnoiti* tachyzoites to induce NETosis in bovine PMN, we analyzed parasite-confronted PMN for NET formation by SEM and immunofluorescence analysis. SEM showed that *B. besnoiti* tachyzoite-exposed PMN indeed released NETs in comparison to unstimulated PMN (**Figures 1A, B**) with several tachyzoites being firmly entrapped by these structures (**Figures 1C, D**; arrows). Of note, the aberrant morphology of entrapped tachyzoites in **Figure 1D** strongly suggested that these stages were dead. To confirm NET identity, classical proteins of NETs were visualized by immunostaining. Here, co-localization of extracellular DNA with histone (i.e., H1) and neutrophil elastase were verified for *B. besnoiti* tachyzoite*-*driven NETs. Some of these NET fibers were in direct contact with *B. besnoiti* tachyzoites, entrapping the parasites (**Figures 1E, F**).

### 3.2 Exposure to *B. besnoiti* tachyzoites induces oxidative responses in bovine PMN

**Figure 2:**
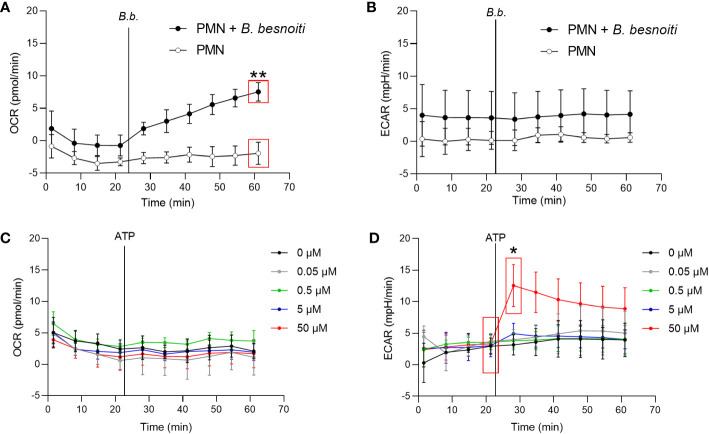
Exposure to *B. besnoiti* tachyzoites induces oxygen consumption in bovine PMN. In the absence of CO_2_, 2x10^5^ PMN were incubated in XF RPMI media for 45 min. Four basal measurements were made and then either *B. besnoiti* tachyzoites **(A, B)** or ATP (0.05-50 µM; **(C, D)** was supplemented at the time point indicated by a vertical line. OCR **(A, C)** and ECAR **(B, D)** values were obtained by Seahorse technology and plotted over time (*n* = 3, for each condition). All data are shown as mean ± SD; *p*-values were calculated by unpaired two-tailed t-test analysis (*n* = 3).

**Figure 3:**
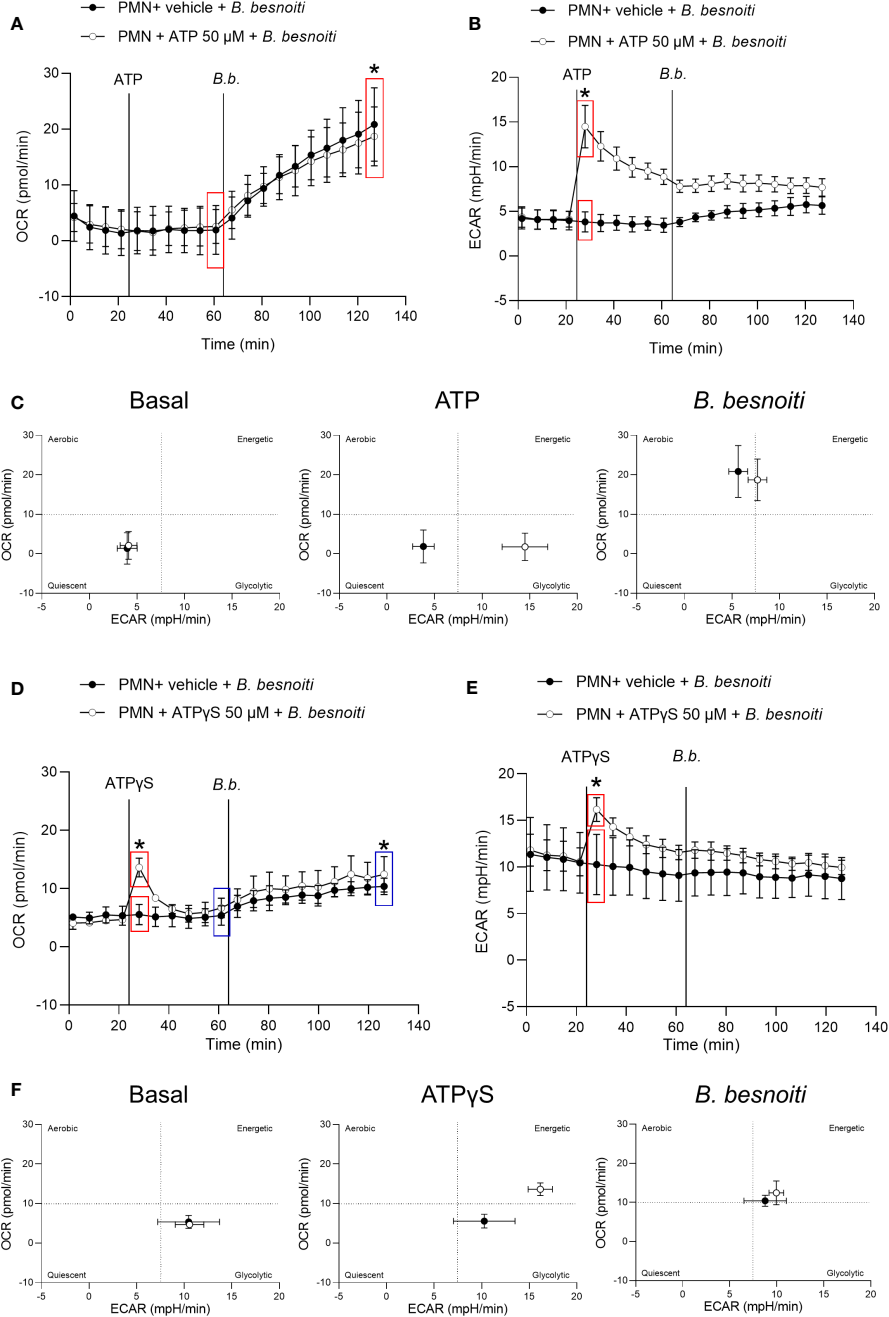
Exposure to *B. besnoiti* tachyzoites induces a metabolic shift toward aerobic carbohydrate catabolism in bovine PMN. After basal measurements, PMN were treated with ATP, ATPγS (50 µM), or vehicle, followed by supplementation of *B. besnoiti* tachyzoites as indicated by vertical lines. Differences in OCR and ECAR from means in the same curve and sharing boxes of the same color were calculated **(A, B, D, E)**. Energetic maps **(C, F)** were generated by presenting OCR (Y-axis) and ECAR (X-axis) as means of the different measurements over time. All data are shown as mean ± SD; *p*-values were calculated by unpaired t-test (A, B, *n* = 6; D, E *n* = 3).

To explore parasite-driven changes in the energetic status and oxidative responses of *B. besnoiti* tachyzoite*-*confronted PMN, we analyzed PMN metabolic parameters by means of oxygen consumption rates (OCR) and extracellular acidification rates (ECAR) (**Figure 2**). *B. besnoiti* tachyzoite supplementation to PMN induced a significant increase in OCR (*p* < 0.01), whilst ECAR was not affected (**Figures 2A, B**). Given that ATP is involved in PMN metabolic and signaling responses, the effect of non-modified ATP supplementation at increasing concentrations was evaluated. Overall, ATP supplementation failed to change oxidative responses (OCR) (**Figure 2C**) but led to a significant (*p* < 0.05) increase in ECAR at a concentration of 50 µM (**Figure 2D**). Based on these data, the influence of ATP on *B. besnoiti* tachyzoite*-*induced activation of PMN was evaluated on the level of additive effects. Therefore, PMN were pre-treated with ATP (50 µM) and then exposed to *B. besnoiti* tachyzoites (PMN: tachyzoites, 1:6). However, ATP pretreatments neither changed the parasite-driven increase in OCR nor ECAR levels (**Figures 3A, B**). A rapid increase in ECAR after ATP supplementation (**Figure 3B**, unfilled circles) was observed when compared to plain XF RPMI medium (**Figure 3B**; filled black circles, *p* < 0.05). Energetic mapping (**Figure 3C**) indicated that *B. besnoiti* tachyzoite exposure induced a shift toward aerobic metabolism in PMN (**Figure 3C**), whilst ATP supplementation rather shifted PMN into a glycolytic status (**Figure 3C**). When the non-hydrolyzable variant of ATP (ATPγS; **Figures 3D, E**) was used, a modest but significant increase in PMN OCR and ECAR was observed, indicating that PMN are activated and move into an energetic status. Again, the addition of ATPγS did not alter the response to *B. besnoiti* tachyzoites (**Figures 3D, E**). Overall, the tachyzoite-induced OCR increase was neither changed by ATP nor by ATPγS supplementation, thereby suggesting a pivotal role of oxygen consumption in parasite-driven activation of bovine PMN.

### 3.3 *B. besnoiti* tachyzoite exposure does not affect total ATP concentration in bovine PMN

**Figure 4:**
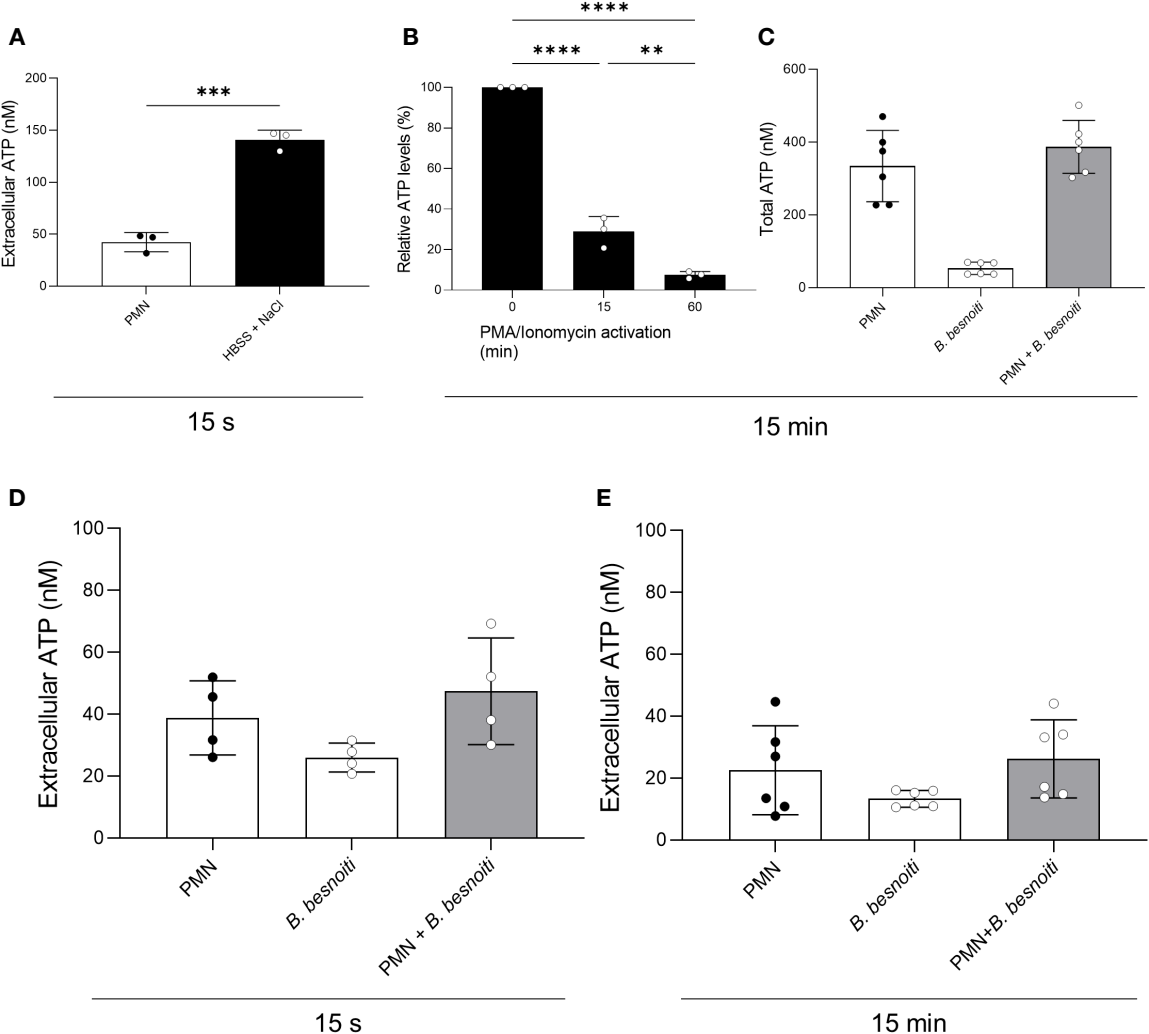
Total PMN ATP and extracellular ATP concentration in *B. besnoiti* tachyzoite-PMN co-cultures. **(A)** For positive control of ATP release, PMN were treated for 15 s with a hypertonic buffer (HBSS supplemented with 1 M sodium chloride). ***p < 0,001. **(B)** For positive control for PMN ATP consumption, PMN were stimulated with PMA/ionomycin (100 nM/5 µM) for 15 and 60 min. **p < 0,01; ****p < 0,0001. **(C–E)** To assess parasite-driven effects on PMN ATP release and consumption, bovine PMN were confronted with *B. besnoiti* tachyzoites (1:6) for 15 s and 15 min. ATP in PMN (total ATP) or supernatants (extracellular ATP) was measured with a commercial kit. All data are shown as mean ± SD; *p*-values were calculated by unpaired two-way t-test **(A)**, or one-way ANOVA followed by Tukey´s multiple comparison **(B-E)** analysis (A, B, *n* = 3; D, *n* = 4; C, *n* = 6).

To investigate a potential change in extracellular ATP and total PMN ATP levels in tachyzoite-PMN co-cultures, ATP concentrations were analyzed at 15 s or 15 min of confrontation by luminometry (**Figure 4**). For positive controls, stimulation of PMN with hypersaline buffer and PMA/ionomycin were used. Exogenous ATP levels were measured in supernatants of PMN treated with a hypertonic buffer for 15 s and indeed showed a significant increase (*p* < 0.001) in extracellular ATP (**Figure 4A**). Furthermore, total ATP levels of bovine PMN were estimated in PMN stimulated with PMA and ionomycin for 15 and 60 min and revealed a continuous drop in ATP levels signifying ATP consumption over time (*p* < 0.0001) (**Figure 4B**). However, when PMN were exposed to *B. besnoiti* tachyzoites, neither changes in extracellular ATP concentration nor total PMN ATP concentration were detected (**Figures 4C–E**).

### 3.4 Non-hydrolyzable ATP boosts *B. besnoiti* tachyzoite*-*induced anchored NET formation

**Figure 5:**
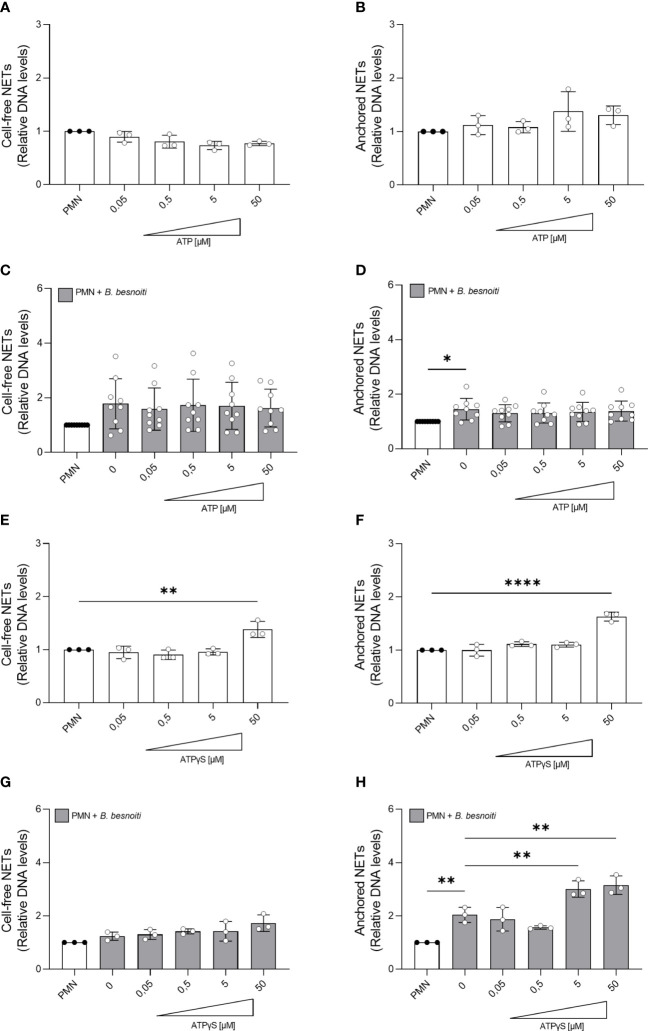
ATPγS supplementation induces NET release and boosts *Besnoitia besnoiti* tachyzoite-driven anchored NET formation. Bovine PMN were pre-treated with increasing concentrations of non-modified ATP or non-hydrolyzable ATPγS (0.05-50 µM) for 10 min. Then, PMN were incubated for 4 h in plain medium **(A, B, E, F)** or exposed to *B. besnoiti* tachyzoites **(C, D, G, H)**. After incubation, extracellular DNA was detected and quantified via picogreen-derived fluorescence intensities using a multi-plate reader at 480 nm excitation/520 nm emission wavelengths. All data are shown as mean ± SD; *p*-values were calculated by one-way ANOVA followed by Dunnett´s multiple comparison test. *p < 0,05; **p < 0,01; ****p < 0,0001.

Given that a high extracellular ATP represents a danger signal and may influence general PMN effector mechanisms, we studied the effects of exogenous ATP on parasite-driven NETosis. NET quantification based on picogreen-derived fluorescence intensities was performed allowing for “cell-free” and “anchored” NET differentiation, thereby reflecting the late phase of NETosis. Therefore, PMN were pre-stimulated with non-modified ATP or ATPγS in a dose-dependent manner and then confronted with *B. besnoiti* tachyzoites (**Figure 5**). As expected, parasite-exposed PMN showed a significant increase in anchored NETs (*p* < 0.05) compared to medium-stimulated controls (**Figure 5D**). Dose-dependent treatments with exogenous non-modified ATP treatment neither affected basal nor tachyzoite-induced NET formation (**Figures 5A–D**). In contrast, ATPγS treatments resulted in a significant increase in basal-anchored (*p* < 0.0001) and cell-free (*p* < 0.01) NET formation in plain PMN at 50 µM concentration (**Figures 5E, G**). Interestingly, in the case of *B. besnoiti* tachyzoite-exposed PMN, ATPγS treatments exclusively induced an increase in the release of anchored NETs but not of cell-free NETs (*p* < 0.01; **Figure 5H**).

### 3.5 P2X1 inhibition by NF449 antagonist blocks *B. besnoiti* tachyzoite*-*induced anchored NET formation

**Figure 6:**
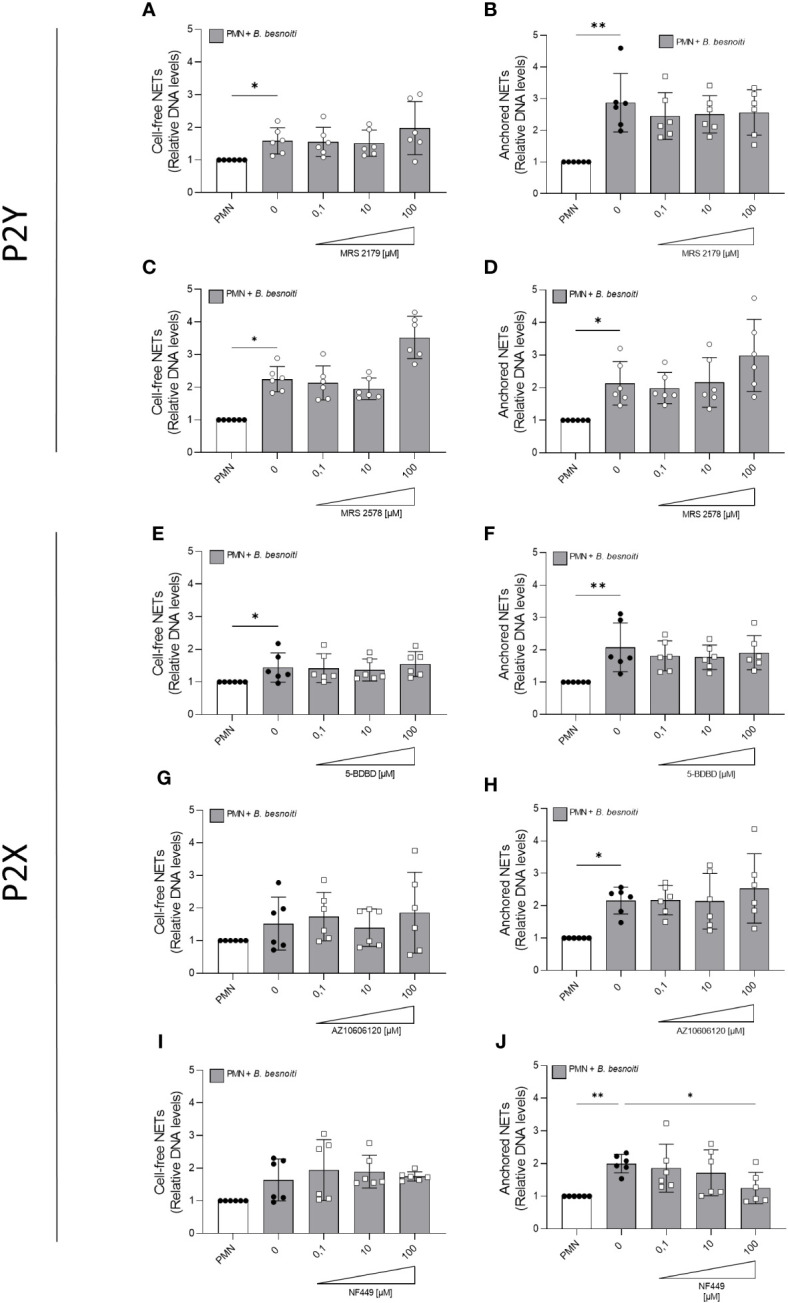
Effects of purinergic receptor antagonist pre-exposure treatments on *Besnoitia besnoiti* triggered NET formation. Bovine PMN were pre-treated for 10 min with increasing concentrations of MRS2179, MRS2578, NF449, 5-BDBD, and AZ10606120 (0.1-100 µM) targeting P2Y1, P2Y6, P2X1, P2X4, and P2X7 receptors, respectively, and then exposed to *B. besnoiti* tachyzoites for 4 (h) Thereafter, cell-free **(A, C, E, G, I)** and anchored **(B, D, F, H, J)** NETs were analyzed. For both NET types, extracellular DNA was detected and quantified via picogreen-derived fluorescence intensities using a multi-plate reader at 480 nm excitation/520 nm emission wavelengths. All data are shown as mean ± SD; *p*-values were calculated by Kruskal-Wallis test followed by Dunn´s multiple comparison test (*n* =6). *p < 0,05; **p < 0,01.

**Figure 7:**
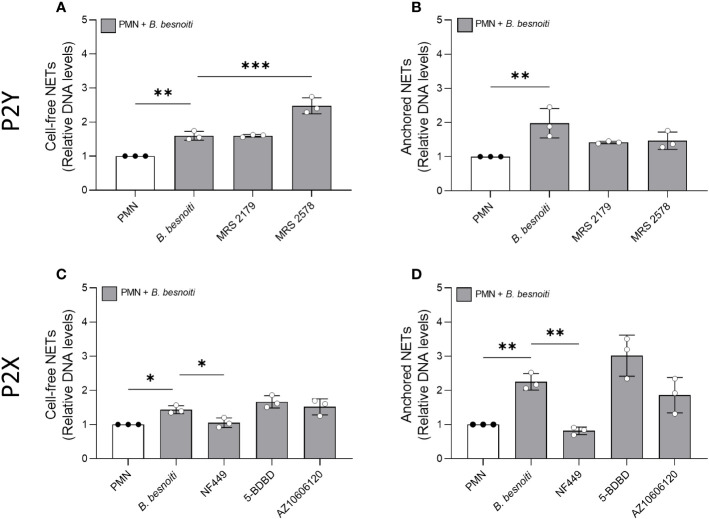
Effects of purinergic receptor antagonist post-exposure treatments on *Besnoitia besnoiti* triggered NET formation. Bovine PMN were first exposed to *B. besnoiti* tachyzoites for 40 min and then treated with 100 µM of MRS2179, MRS2578, NF449, and 5-BDBD targeting P2Y1, P2Y6, P2X1, P2X4, and P2X7 receptors, respectively. After 4 h of incubation, cell-free **(A, C)** and anchored **(B, D)** NETs were analyzed. For both NET types, extracellular DNA was detected and quantified via picogreen-derived fluorescence intensities using a multi-plate reader at 480 nm excitation/520 nm emission wavelengths. All data are shown as mean ± SD; *p*-values were calculated by an ordinary one-way ANOVA with Dunnett´s multiple comparison analysis. (*n* =3). *p < 0,05; **p < 0,01; ***p < 0,001.

Given that exogenous ATP seemed to play a role in NET induction, we further investigated the relevance of purinergic signaling pathways in *B. besnoiti* tachyzoite-induced NETosis. To monitor the role of different purinergic receptors, PMN were pre-treated with receptor antagonists of different P2X (P2X1, P2X4, and P2X7) and P2Y (P2Y1 and P2Y6) receptors at different concentrations (see **Table 1**) and then confronted with parasite stages (**Figure 6**). In antagonist-free controls, PMN confrontation with *B. besnoiti* tachyzoites resulted in a significant increase (*p* < 0.05) in anchored NET formation when compared to controls. In line with previous data (32), NF449 pre-treatments targeting the purinergic P2X1 receptor resulted in a significant decrease in *B. besnoiti* tachyzoite*-*triggered anchored NETs (*p* < 0.05) when compared to non-treated PMN (**Figure 6J**). However, all other treatments (pharmacological inhibition of P2X4, P2X7, P2Y1, and P2Y6 receptors) failed to affect parasite-triggered anchored or cell-free NET formation (**Figures 6A–H**). Besides antagonist pre-treatments, the inhibitors were also added 40 min after *B. besnoiti* tachyzoite exposure to determine if any of the antagonists were capable of reverting parasite-induced NET formation, previously described as a “point of no return” and defined as consumption of intracellular ATP by PMN after 30-40 min of activation (16). In this experimental setting, a selective efficacy of NF449 was confirmed since exclusively this treatment significantly decreased *B. besnoiti* tachyzoite*-*triggered anchored (*p* < 0.01) and cell-free (*p* < 0.05) NETs (**Figures 7C, D**). The sum of these data indicated that tachyzoite-induced NETosis selectively depends on P2X1-mediated signaling.

### 3.6 NF449 blocks *B. besnoiti tachyzoite-*induced NET formation in a dose-dependent manner and without affecting cell viability

**Figure 8:**
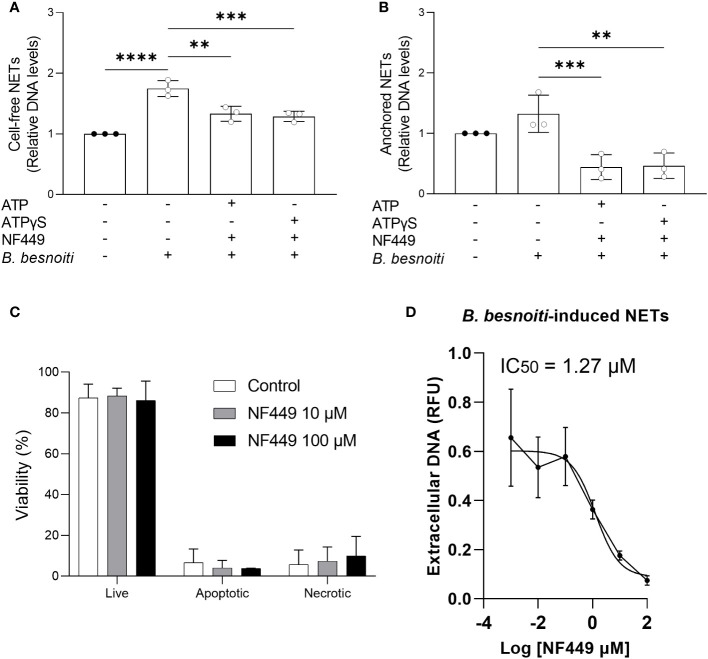
NF449 does not affect PMN’ viability and inhibits *Besnoitia besnoiti* triggered NET formation in an ATP-independent but dose-dependent manner. **(A, B)** Bovine PMN were pre-treated with NF449 (100 µM) in the presence or absence of ATP/ATPγS and then confronted with *B. besnoiti* tachyzoites. After 4 h of incubation, extracellular DNA was detected and quantified via picogreen-derived fluorescence intensities. **(C)** Annexin V-FITC and propidium iodide staining of PMN treated with NF449 for 4 h. **(D)** NF449-based dose-response-inhibition of *B. besnoiti* tachyzoite-induced NET formation. The IC50 was calculated by a nonlinear regression analysis. All data are shown as mean ± SD; *p*-values were calculated by one-way ANOVA with Dunnett´s multiple comparison analysis. (*n* = 3). **p < 0,01; ***p < 0,001; ****p < 0,0001.

Since NF449 was the only inhibitor that consistently reduced *B. besnoiti* tachyzoite*-*induced NET formation, we further studied the dose dependency of these reactions and eventual ATP-driven changes but also controlled the potential effects of this P2X1 antagonist on PMN necrosis and apoptosis by propidium iodide and Annexin V-FITC/PI staining, respectively (**Figure 8**). Overall, the combination of exogenous ATP or ATPγS (50 µM) supplementation and *B. besnoiti* tachyzoite exposure did not change the consistent significant decrease (*p* < 0.01) in anchored and cell-free NET formation driven by NF449 pre-treatments (**Figures 8A, B**). As revealed by propidium iodide and Annexin V-based assays, NF449 treatments (10 µM and 100 µM) had no effect on PMN necrosis or apoptosis since the proportions of vital PMN remained equal (88.4% and 86.1%, respectively) compared to non-treated controls (87.4%) (**Figure 8C**). Analysis on NF449-related dose dependence revealed an IC_50_ of 1.27 µM for *B. besnoiti* tachyzoite*-*induced NET formation (**Figure 8D**). In summary, NF449-mediated inhibition of *B. besnoiti* tachyzoite-induced NET formation was dose-dependent and occurred without affecting PMN viability (**Figure 8**).

### 3.7 *B. besnoiti* tachyzoite exposure induces a P2X1-dependent clustering of bovine PMN

**Figure 9:**
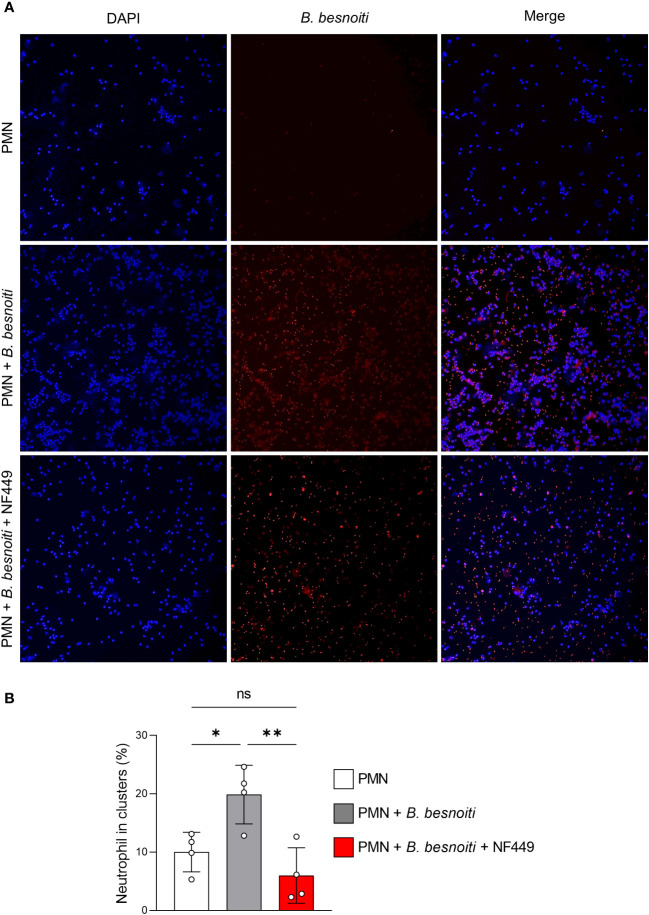
*B. besnoiti* tachyzoite-induced clustering of bovine PMN depends on P2X1-based purinergic signaling. PMN were co-cultured with tachyzoites for 4 h in the presence or absence of NF449 (100 µM). **(A)** Exemplary illustrations of tachyzoite-PMN co-cultures stained for DNA (DAPI, blue) and parasite stages (red). **(B)** DANA-based quantification of cluster formation. All values are presented as mean ± SD, and *p*-values were calculated using one-way ANOVA followed by Tukey´s multiple comparisons. (*n* = 4). *p < 0,05; **p < 0,01.

During co-culture experiments, we noticed that the presence of *B. besnoiti* tachyzoites seemed to drive PMN clustering. To follow this impression and to study whether this finding depended on purinergic signaling, we analyzed parasite-mediated PMN cluster formation in the presence and absence of the P2X1 antagonist NF449 (**Figure 9**). Indeed, an increase (*p* < 0.05) in PMN participating in clusters was verified for *B. besnoiti tachyzoite-*confronted PMN after 4 h of incubation when compared with parasite-free controls (**Figure 9B**). Notably, the number of PMN participating in clusters decreased to basal levels (*p* < 0.01) when PMN were pre-treated with the P2X1 inhibitor NF449 at a concentration of 100 µM (**Figure 9B**), thereby confirming the critical role of purinergic signaling in the clustering process.

## 4 Discussion

In the present study, we studied the role of ATP and purinergic receptor signaling in *B. besnoiti* tachyzoite-induced bovine PMN activation with respect to *i)* PMN energetic state, *ii)* NETosis, and *iii)* PMN clustering. Since the discovery of NETs, there is accumulating evidence that NET formation represents a conserved mechanism among multiple kingdoms (45–47). Referring to protozoan parasites, NETosis is efficiently induced by both extra- and intracellular parasites, such as *T. b. brucei*, *T. gondii, E. bovis, C. parvum, N. caninum*, and *B. besnoiti* (21, 24, 26–28, 31–33, 43, 48–51). In the current study, we confirmed the PMN release of DNA-rich extracellular structures decorated with H1 and NE in response to vital *B. besnoiti* tachyzoites by SEM and immunofluorescence. The current data are in line with previous reports on NETs triggered by *B. besnoiti* tachyzoites (30–32, 52, 53). Notably also, other innate cell types like primary bovine monocytes reacted to (M)ETosis when being exposed to *B. besnoiti* tachyzoites (53), thereby emphasizing the general capacity of these parasitic stages to induce this innate effector mechanism and enhancing the likelihood of its occurrence *in vivo* during bovine besnoitiosis.

PMN are considered as highly glycolytic cells and glycolysis is active when PMN are producing ROS or performing phagocytosis (54, 55). Recently, gluconeogenesis and glycogenesis were demonstrated as critical for PMN’ lifespan and function, indicating a complex and context-dependent PMN catabolism to fulfill killing functions (56). In general, PMN-derived oxidative responses (here: OCR) are mainly derived from NOX-based ROS production, with little or no mitochondrial contribution at all (57, 58). In the present study, the early activation of bovine PMN seemed to be related to ROS production without the involvement of glycolysis. Thus, PMN oxidative activity increased at 5-10 min post-*B. besnoiti* tachyzoite exposure, as detected by a continuous increment of OCR values most likely reflecting PMN oxidative burst. This is in line with previous observations using DCFH-DA to measure *B. besnoiti* tachyzoite*-*induced ROS in PMN (30). These data, even though measured by different techniques, confirm a consistent role of ROS production, accompanied by oxygen consumption in parasite-induced NETs as driven by *T. gondii* (49), *E. bovis* (42), and trypomastigote stages of *T. b. brucei* (28). Overall, *B. besnoiti* tachyzoite*-*induced oxygen consumption was not influenced by the addition of exogenous ATP or ATPγS, thereby denying a priming effect of ATP. Intriguingly, *B. besnoiti* tachyzoite*-*induced NET formation was recently proven as mitochondrial ATP synthase-dependent since it was significantly dampened by oligomycin treatments (32), indicating a potential later relevance (i.e., after 30 min of confrontation) of the mitochondrial activity or mitochondrial ROS to sustain *B. besnoiti* tachyzoite*-*induced NET formation. Given that ECAR enhancement mirrors the extracellular accumulation of lactate (58), the current data may indicate that immediate bovine PMN reactions to parasite stages may not rely on glycolytic responses. On the other hand, ATP/ATPγS supplementation led to an acute (within a few minutes) increase in PMN ECAR. However, when referring to later effector phases by analyzing NET formation after 6 h of co-culture, metabolic signatures of *B. besnoiti* heat-inactivated tachyzoite-exposed bovine PMN showed a significant increase in glucose and serine consumption and glutamate release in addition to a decrease in glutamine release during NETosis, thereby suggesting a switch in parasite-driven metabolic responses toward glycolysis (32). The differences in the current data may rely on the use of heat-inactivated tachyzoites, the presence of cytochalasin to block phagocytosis - likely altering PMN catabolism - and on the later time point compared with the current data (40 min vs 6h), highlighting again the time- and context-dependent nature of PMN responses upon activation (56). Recent evidence on PMN-specific immunometabolism revealed that this innate “differentiated” cell type can indeed selectively induce different metabolic pathways after activation, depending on the effector mechanism to be performed (e.g., chemotaxis, ROS production, NET formation, or degranulation) (59). Overall, in the case of NET formation, there is a general consensus that glycolysis seems to be the main mechanism of energy generation in PMN. However, referring to *B. besnoiti* tachyzoite-driven NETosis (32), the blockage of glycolysis via FDG treatments did not directly affect NET formation. Nevertheless, a key role of pyruvate- and lactate-mediated metabolic pathways for proper tachyzoite-mediated NETosis was demonstrated since chemical inhibition of lactate release via oxamate and dichloroacetate and blockage of pyruvate dehydrogenase, α-ketoglutarate dehydrogenase, and transketolase via oxythiamine indeed dampened *B. besnoiti* tachyzoite-driven NETosis (32). Comparable NET-related findings were reported for the closely related apicomplexan parasite *E. bovis*. Here, *E. bovis*-triggered NET formation was also not affected by FDG treatments, but by oxamate, oligomycin, and MCT inhibitors (42). Altogether, these data may indicate a pivotal role of secondary metabolites of the carbohydrate catabolism rather than of glycolysis itself in apicomplexan parasite-induced NET formation at times later than 40 min of activation (32, 42).

In general, ATP is either synthesized by mitochondrial respiration or glycolysis. Importantly, ATP not only represents the key energy source of metabolism but also actively participates in the activation of PMN effector function via purinergic signaling. During inflammation or ischemia, several cell types release cellular ATP as a danger and “find me” signal fueling inside-out signaling mechanisms that regulate the activation and function of PMN. Under normal circumstances, the released ATP boosts PMN effector functions like chemotaxis, degranulation, phagocytosis, and NETosis through autocrine feedback mechanisms that involve ATP and adenosine receptors (60, 61). These purinergic signaling mechanisms regulate calcium influx and other downstream signaling pathways that are required for proper PMN functionality. Overall, a complex network of metabolic pathways regulates ATP release and purinergic signaling mechanisms. This network involves mitochondria that produce the ATP which fuels purinergic signaling. Thus, mitochondria are the link between metabolic and calcium signaling events and the purinergic signaling mechanisms that regulate immune cell functions. Pre-treatments of bovine PMN with oligomycin (blocking mitochondrial ATP synthase) entirely abolished *B. besnoiti* tachyzoite-induced cell-free NET formation, thereby indicating the relevant role of mitochondrial ATP production and purinergic signaling for effective parasite-driven NETosis. In order to study the paracrine effects of ATP, we quantified both extracellular and total ATP concentrations in cultures of *B. besnoiti* tachyzoite*-*confronted PMN. In supernatants of unstimulated control PMN, an extracellular ATP concentration ([ATP]) of 27.3 nM and 22.6 nM was detected after 15 s and 15 min, respectively. Moreover, a total cellular [ATP] of 334.2 nM (334 fmol/cell) was measured in untreated bovine PMN. In human PMN, the reported ATP intracellular concentration is 1.9 fmol/cell (55). In contrast, under physiological conditions, extracellular [ATP] is typically low (< 1 µM) (62, 63). Whilst current data on extracellular [ATP] match typical basal concentrations, current total cellular [ATP] values seemed rather high; however detailed reference data for bovine PMN are currently missing in the literature. Interestingly, recent data proved that PMN express Cx43 and Panx1 hemi-channels mediating ATP release linked to autocrine purinergic signaling which finally regulates PMN chemotaxis (61). In this context, supplementation of exogenous non-hydrolyzable ATP (ATPγS), but not of non-modified ATP, significantly induced cell-free and anchored NET formation. Moreover, ATPγS supplementation enhanced anchored NET formation in *B. besnoiti* tachyzoite*-*exposed PMN. In general, ATP levels of the extracellular environment are tightly controlled and eventually lowered by the conversion of different cell types that express plasma membrane ectonucleotidases, such as nucleoside triphosphate diphosphohydrolase 1 (CD39, convert ATP/ADP to AMP) and ecto-5′-nucleotidase (CD73, convert AMP to ADO). Notably, CD39 and CD73 are both expressed in PMN (64). Thus, these ectonucleotidase activities of bovine PMN may explain both the failure of non-modified ATP supplementation to induce NETs and the potential of ATPγS (which cannot be hydrolyzed by ectonucleotidases) to successfully trigger this effector mechanism.

Recent studies have highlighted the critical role of autocrine purinergic signaling in different PMN-derived effector mechanisms. Two main classes of membrane receptors mediate the effects of extracellular ATP: metabotropic G protein-coupled P2Y receptors and ionotropic P2X receptors acting as ligand-gated non-selective cation channels. For P2Y receptors, eight subtypes (P2Y1/2/4/6/11/12/13/14) are known, whereas, for P2X, seven subtypes (P2X1–7) have been found (64, 65). Based on data from mRNA, protein, and functional assays, PMN have been reported to express P2X1, P2X7, P2Y2, and P2Y14, as well as all four ADO receptors (61). In this context, Panx1 hemi-channels rapidly release ATP during PMN chemotaxis from pseudopod protrusions, amplifying chemotactic signals through activation of P2Y2-mediated mTOR signaling at the leading edge (66–68). Furthermore, PMN stimulation with LPS-enhanced phagocytosis of *Escherichia coli* in humans, and these effects were abolished when the P2X1 purinergic receptor was blocked (69). Moreover, an antagonist of P2Y6 suppressed monosodium urate crystal-induced PMN oxidative burst (35, 70) and aggregated NET formation (70). In the current study, the effects of different P2X and P2Y receptor antagonists (MRS2179, MRS2578, NF449, 5-BDBD, and AZ10606120) on tachyzoite-driven NET formation were explored. We showed that exclusive pre-treatments of PMN with the P2X1 antagonist NF449 resulted in a significant reduction of parasite-induced anchored NET formation whilst all other antagonists failed to affect NETosis. Similar results were obtained when applying post-exposure treatments. The NF449-related data in principle confirm the recent findings of Zhou et al. (2020) stating the role of P2X1-mediated purinergic signaling in *B. besnoiti* tachyzoite*-*mediated NETosis. We furthermore proved that NF449 treatments neither induced apoptotic nor necrotic PMN cell death and elucidated dose-dependent effects of NF449 by estimating an IC_50_ of 1.27 µM. Given that NF449 treatments also inhibited NET formation induced by *T. b. brucei* (28) and *C. parvum* (43) stages, a conserved P2X1-related mechanism in parasite-driven NETosis may be proposed. As an additional and interesting finding, P2X1-mediated signaling also seemed involved in *B. besnoiti* tachyzoite-mediated clustering of bovine PMN. During PMN-tachyzoite-co-cultures, we consistently observed that PMN tend to form clusters which most probably result from chemotactic and migratory actions. However, pre-treatments of PMN with NF449 significantly diminished the number of PMN forming clusters. Therefore the current study adds new data on the role of P2X1-mediated purinergic signaling in parasite-PMN interactions and expands its importance to further PMN-derived effector mechanisms than NETosis. Future studies will focus on its role in other tachyzoite-driven PMN mechanisms.

## Data availability statement

The raw data supporting the conclusions of this article will be made available by the authors, without undue reservation.

## Ethics statement

The animal study was approved by Ethic Commission for Experimental Animal Studies of the Federal State of Hesse (Regierungspräsidium Giessen; GI 18/10 Nr. V 2/2022; JLU-No. 0002_V) and are in accordance to European Animal Welfare Legislation: ART13TFEU and currently applicable German Animal Protection Laws. The study was conducted in accordance with the local legislation and institutional requirements.

## Author contributions

AT, CH, and IC: conceptualization and supervision. GE: investigation (PMN isolation, *Besnoitia besnoiti*/MDBK cell culture, Seahorse, Flow cytometry, ATP measurement, and extracellular DNA quantification experiments), formal analyses, data visualization, and writing the original draft. IC: formal analyses and data visualization. LR: obtained epifluorescence microscopy images. GE, IC, LR, CH, and AT: reviewed the manuscript. CH and AT: funding acquisition. All authors contributed to the article and approved the submitted version.
